# The making of accessible Android applications: an empirical study on the state of the practice

**DOI:** 10.1007/s10664-022-10182-x

**Published:** 2022-08-06

**Authors:** Marianna Di Gregorio, Dario Di Nucci, Fabio Palomba, Giuliana Vitiello

**Affiliations:** University of Salerno, Fisciano, Italy

**Keywords:** Mobile accessibility, Mobile app evolution, Universal design

## Abstract

Nowadays, mobile applications represent the principal means to enable human interaction. Being so pervasive, these applications should be made usable for all users: accessibility collects the guidelines that developers should follow to include features allowing users with disabilities (e.g., visual impairments) to better interact with an application. While research in this field is gaining interest, there is still a notable lack of knowledge on how developers practically deal with the problem: (i) whether they are aware and take accessibility guidelines into account when developing apps, (ii) which guidelines are harder for them to implement, and (iii) which tools they use to be supported in this task. To bridge the gap of knowledge on the state of the practice concerning the accessibility of mobile applications, we adopt a mixed-method research approach with a twofold goal. We aim to (i) verify how accessibility guidelines are implemented in mobile applications through a coding strategy and (ii) survey mobile developers on the issues and challenges of dealing with accessibility in practice. The key results of the study show that most accessibility guidelines are ignored when developing mobile apps. This behavior is mainly due to the lack of developers’ awareness of accessibility concerns and the lack of tools to support them during the development.

## Introduction

Mobile applications, a.k.a. apps, are nowadays used by billion users for any social and emergency connectivity (Wasserman 2010). The trend is tremendously and continuously increasing these days: the rise of social distancing has indeed changed the way people communicate and interact with each other (Martin et al. 2016; Statista 2020). In such a context, mobile apps represent one of the primary means of allowing human interaction. Therefore, an ever-increasing population of users needs to interact with the functionalities they implement. This aspect does not only represent a challenge for researchers in the field of computer-human interaction (CHI) but also for software maintenance and evolution research, which is called to devise novel instruments to support developers when evolving successful mobile apps that all types of users can use (Martin et al. 2016; Yan and Ramachandran 2019).

Applications that are not accessible or are only partially accessible are an obstacle for both individuals and businesses (Ballantyne et al. 2018; Yan and Ramachandran 2019). For a single user, a hard-to-use app will either be a source of stress and frustration or be entirely sidelined in favor of a more accessible alternative (Sevilla et al. 2007). For a business, the fewer users can use their mobile application, the lower the translated revenue stream will be (Wentz et al. 2017). The pervasiveness of mobile applications has led researchers to reason more and more in terms of *accessibility*. This trend is giving rise to a research field that aims at developing mobile apps usable by those affected by disabilities (e.g., visual impairments) (Leporini et al. 2012; Vitiello et al. 2018), which represent over one billion (around 15%) of the world’s population. Ensuring the accessibility of the app functionalities has become more crucial than ever (Yan and Ramachandran 2019) when people affected by disabilities are more dependent on their mobile devices.

The two main operating systems for tablets and smartphones, i.e., iOS and Android, are equipped with pre-installed accessible functions, including screen reading functionalities as in the case of TalkBack for Android. The unique needs of individuals with disabilities and their right to participate in the digital age cannot be ignored by developers. However, differently from iOS, accessibility work in Android apps is very limited (Martin et al. 2016; Yan and Ramachandran 2019) and, as such, it is unclear to what extent developers implement universal design principles or use accessibility features in their mobile applications.

So far, most of the research on accessibility has focused on the web and mainly provided guidelines and instruments that developers can employ to implement accessible websites (e.g., Flatla 2011; Friedman and Bryen 2007; Sevilla et al. 2007). On the contrary, the accessibility of mobile applications has not been examined so thoroughly (Yan and Ramachandran 2019) and, as a matter of fact, it still represents an open research challenge to face.

In the recent past, empirical investigations have been conducted to study how developers discuss the matter on StackOverflow (Vendome et al. 2019) and how existing accessibility features support users with disabilities (Kocieliński and Brzostek-Pawłowska 2013; Walker et al. 2017). Nevertheless, there is still a notable lack of knowledge on the way developers approach the problem of accessibility and whether they implement the available guidelines to develop accessible applications. An improved understanding of these aspects is crucial to guide future software maintenance and evolution research efforts toward the definition of design, evolutionary, and testing techniques that can better support practitioners while developing mobile accessible applications.

In our previous registered report (Di Gregorio et al. 2020),^1^ we designed an exploratory empirical investigation into the making of mobile apps from the perspective of accessibility to bridge the current gap of knowledge concerning the relation between mobile apps and accessibility. We focused on Android for a two-fold reason. On the one hand, it has been the subject of previous accessibility studies. On the other hand, although a vast number of apps is developed worldwide on this platform, still little is known on how to best engineer the problem in Android devices—as opposite to iOS and Apple, which provide an integrated set of devices and features to handle accessibility (Darvishy 2014). More particularly, we discussed our plan toward this goal by defining two research questions to understand (i) whether and to what extent the available accessibility guidelines are implemented in Android applications and (ii) the developer’s opinions about the matter. We sought to elicit the state of the practice and the key issues and challenges faced by developers when dealing with accessibility.

In this paper, we follow up on the registered report and present the results of our study. The study has adopted a two-step methodology. We first conducted manual coding activities to quantify how existing accessibility guidelines are implemented in the context of 50 top-rated Android applications. Then, we conducted a survey study with 70 mobile developers and ten semi-structured interviews to gather insights into the issues and challenges of developing accessible apps and understand the extent to which developers are implementing accessibility support in Android apps.

The key results yielded by our study are that only a subset of the available guidelines is typically implemented in Android apps, and these mainly relate to aspects like color contrast and interactive content. While surveying developers, we could recognize a general lack of awareness of accessibility concerns; furthermore, developers indicated the lack of (semi-)automated support to control accessibility while developing mobile applications.

The findings of the paper allow us to provide the research community with a set of open issues and challenges that represent the next research avenues that should be addressed to provide developers with usable accessibility tools.

To sum up, our study provides the following contributions:

1. An empirical study reporting the accessibility guidelines that are and are not implemented in Android applications, which can be used by researchers and tool vendors as a basis for either prioritizing accessibility concerns within techniques/tools or conducting further analyses into the specific reasons why certain guidelines are more/less applied in practice;
2. Insights into the developer’s perception that researchers can use to understand the underlying motivations leading practitioners not to apply accessibility guidelines as well as by tool vendors to tune current tools based on the opinions that developers have of specific accessibility concerns;
3. A list of current issues and challenges that developers face when dealing with accessibility in practice that can be useful for researchers to motivate and conduct additional studies into the matter;
4. A publicly available replication package (Di Gregorio et al. 2021) containing the data to address the goals of our study—data are anonymized whenever needed. The package includes a novel dataset reporting the accessibility guidelines implemented in a set of 50 Android apps— researchers can use it as ground truth to evaluate novel accessibility tools.

### Structure of the Paper

Section 2 discusses the background on accessibility guidelines and overviews the related literature. In Section 3 we describe the research questions and methodology employed to address our study, while Section 4 reports the achieved results. In Section 5, we summarize the main findings, discuss the limitations of the study, and outline the key implications that our work has for the research community. Section 6 overviews and discusses how we mitigated possible threats to validity. Finally, Section 7 concludes the paper and presents our future research agenda on the matter.

## Background and Related Work

In this section, we define accessibility, provide an overview of the currently available guidelines to create accessible apps, and discuss the related literature, comparing it with the methodology employed in our study.

### Accessibility: Definition and Guidelines Overview

According to Iwarsson and Ståhl (2003), accessibility is defined as *“the quality of being easily reached, entered or used by people with disabilities”*. Mobile accessibility refers to making websites and apps more accessible to people with disabilities when using smartphones and other mobile devices (W3C 2020).

While the definition explicitly targets people suffering from disabilities, it is worth mentioning that accessibility is a desirable property for other groups of people as well, since these could also benefit from the availability of accessibility functionalities (Lawrence and Giles 2000). As such, we could generalize the concept of accessibility and state that it is the practice of making websites and apps usable *by as many people as possible* (Lawrence and Giles 2000). Indeed, there are two key elements driving accessibility: (1) The attention to the problems of accessing websites and apps for people with disabilities; and (2) The attention to guaranteeing *universality* of access, that is, not to exclude anyone: not only people with disabilities in the strict sense but, for example, also those suffering from temporary disabilities, those with obsolete equipment or slow connections.

Various mobile accessibility standards have been proposed, including those defined by W3C (W3C 2020) and by the UK BBC Standards and Guidelines for Mobile Accessibility (BBC 2021). Within these standards, several recommendations have been formulated to provide better support for people with different types of disabilities, including motor, hearing, and vision problems. Several companies have also created their list of developer guidelines based on standards such as the Android Accessibility Developer Guidelines (Android 2021), Apple’s Accessibility Developer Guidelines (Apple 2021) and the IBM accessibility checklist (IBM 2020). In our work, we focused on accessibility issues in Android apps and considered the recommendations provided by Android, W3C, and BCC.

Standard controls, objects, and elements should be used to ensure a higher level of accessibility as custom controls tend not to implement accessibility as fully as standard platform controls. When standards and guidelines are implemented using non-standard techniques, there is a risk that users who depend on platform-specific accessibility features such as accessibility settings and speech output are excluded from accessing the content.

Progressive enhancement is recommended to ensure that users with accessibility settings or assistive technology enabled using older phones and platforms can access the content. This mechanism ensures that the content and features are accessible even if the experience is slightly altered. All content must be accessible and navigable using the platform navigation paradigm for assistive technology. For example, the directional controller must be supported on Android to allow TalkBack screen reader users to review and navigate the page content. Android requires that all elements be accessible from the keyboard to be accessed with a D-pad or trackball. In this respect, Android 4.0 has reduced this requirement somewhat by including an *“Explore by touch”* method.

When applications or sites block, disable, or interfere with platform-specific accessibility features or technology, users with disabilities may not be able to use the site or the app. Potential problems include zoom suppression, garbled screen content, or the inability to run assistive technology. This behavior could occur when the technology directly controls audio, video, or CPU resources preventing assistive technology from accessing these resources promptly.

Some users with disabilities may require more accessibility features because they may have more disabilities. For example, a user may be deaf and blind or may have poor eyesight and may be unable to use a pointing device or touchscreen. More modes of operation should be supported that allow users to access content according to their preferences. On Android, for example, built-in keyboard support should not prevent other standard touch events.

### Accessibility: State of the Art

The topic of accessibility is rapidly gaining interest in software engineering and closely related research communities, like computer-human interaction and computer-supported cooperative work. These multi-disciplinary research opportunities allow for treating the problem from different perspectives. However, although accessibility has long been investigated in the context of web applications (Harper and Chen 2012; Sierkowski 2002; Sloan et al. 2006), and many books are now available to drive developers toward the construction of accessible websites (Harper and Yesilada 2008; Paciello 2000; Rutter et al. 2007), the definition of accessibility principles and guidelines for mobile applications can be still considered a work in progress.

From an empirical standpoint, Kocieliński and Brzostek-Pawłowska (2013) investigated the currently available accessibility features with virtual QWERTY keyboards on mobile devices, comparing them against the use of an integrated Braille notetaker. The controlled study involved visually impaired users who were called to complete tasks using the two treatments. The main findings reported were that the existing mobile support is not sufficient to assist visually impaired users. Indeed, the integration of a Braille notetaker led to better results in terms of task completion time. Zhong et al. (2015) augmented the touch feature of Android devices in order to facilitate less precision to a targeted location and assist with gestures that might not be suitable for individuals with tremors, e.g., double-tapping the same location. Mehta et al. (2016) conducted an empirical study—involving 12 blind users—to study the accessibility of date-pickers and proposed to augment the current capabilities of mobile devices with additional features able to support blind users. On a similar note, Xie et al. (2015) focused on the understanding and improvement of the support provided for GUIs responsiveness when connecting smartphones to external displays, while Milne et al. (2014) studied the accessibility of mobile health sensors for blind users: interestingly, they found that most of the accessibility problems identified could be solved with a small amount of effort. Conversely, another interesting and little investigated problem faced by Ichioka et al. (2020) is the percentage of malware in apps that use accessibility services that is constantly increasing. Therefore, in the future it is necessary to investigate the identification of specific countermeasures for malware using accessibility services.

Case studies have also been performed to study specific types of disabilities. Serra et al. (2015) assessed four Brazilian government applications against the W3C guidelines, discovering that most of them were not applied. In this scenario, Quispe et al. (2020) investigated the prioritization of mobile accessibility guidelines extracted from e-MAG (the Brazilian Government Accessibility Model) to help dealing with limited resources while also addressing accessibility. Walker et al. (2017) evaluated several weather apps and their usability/accessibility for blind and sighted users: as a result, they discovered that most of the considered apps were not designed to be universally accessible. Al-Subaihin et al. (2013) reported that, if appropriately used, structural HTML elements can make the functionality of TalkBack and VoiceOver similar in mobile web apps and native applications. Also, Krainz et al. (2018) proposed a change in the mobile app development to support accessibility, concluding that a model-driven approach with automated code generation might potentially avoid many of the accessibility problems experienced by visually impaired users. Eler et al. (2019) analyzed comments made at the Google Play Store and FDroid, aiming to identify whether users comment about accessibility problems. When they evaluated the ratings of the apps, they noticed that users generally do not mention accessibility issues in their reviews.

Some more recent studies focused on defining specific instruments and methods to support users with special needs. For instance, Araújo et al. (2017) defined a manual test that can assess whether mobile audio games meet the need of visually impaired users. Similarly, Díaz-Bossini et al. (2014) and Díaz-Bossini and Moreno (2014) proposed guidelines to make mobile applications closer to the needs and requirements of older users. Park et al. (2014) and Ross et al. (2018) analyzed the image-based button labeling problem by focusing on missing and alternative text labels, respectively. Subsequently, again Ross et al. (2020) performed a large-scale analysis of free Android apps, exploring the frequency of accessibility barriers and the factors that may have contributed to barrier prevalence. They tested a population of 9,999 apps but limited themselves to just testing seven accessibility barriers. Our study does not analyze a large-scale app population but we tested all accessibility guidelines identified.

When turning the attention to the software engineering research community, Armaly and McMillan (2016), Armaly et al. (2017, 2018) conducted several studies aiming at comparing program comprehension tactics applied by blind and sighted programmers. Their key findings reported that, despite having different reading processes, both tend to prioritize the understanding of method signatures; furthermore, audio highlight facilities might provide additional support to blind programmers when skimming code on the web. McMillan and Rodda-Tyler (2016) also reported on a didactic software engineering course setting that allows blind and sighted programmers to collaborate more effectively and improve their capabilities to share programming expertise and knowledge.

The above-mentioned papers are different from the study proposed herein. Most of them focused on accessibility from the perspective of specific users and aim at characterizing the limitations of currently available guidelines compared to the needs and requirements of such users. On the contrary, our focus is on developers and how they act when it turns to keep accessibility into account, highlighting the challenges they face when implementing accessibility guidelines and the additional instruments that they would need to build more accessible mobile applications. The preliminary results of de Almeida and Gama (2021) show, in fact, that developers have a worse perception than interface designers on this topic.

Vendome et al. (2019) first performed a mining study showing the limited usage of accessibility APIs in a set of 13,817 apps. Then, they focused on the developer’s perspective by mining StackOverflow posts related to accessibility. From this analysis, they identified the aspects that developers mainly implement in their apps and those requiring more effort. Compared to that study, ours can be seen as complementary, mainly because we adopted a mixed-research method that allowed us to gain more precise information on the extent to which accessibility features are applied during development.

The closest work is that of Alshayban et al. (2020). The authors have conducted an empirical study aimed at understanding the accessibility of the Android apps. They reported a large-scale analysis, however, analysing only 11 accessibility guidelines. The authors also presented the results of a survey to detect current practices and challenges in Android apps with regard to accessibility. Compared to this study, on the one hand, we conducted manual analyses to test a large number of accessibility guidelines and verify which of them are implemented in Android applications. In this way, we can be more precise in indicating the specific guidelines that developers tend to care about more when including accessibility concepts. Furthermore, such an analysis leads to additional insights into whether and how developers implement critical guidelines that are relevant for users (e.g., the *MUST* ones). On the other hand, we directly inquired developers having two key advantages. First, we could ask more complete and specific questions on their perceptions and opinions of accessibility in practice, rather than letting them emerge from the analysis of posts. Second, we could involve a broader population of developers rather than focusing on those subscribed to StackOverflow—which might provide a limited view on the matter.

### Accessibility: State of Practice

When considering the tools to assess accessibility of Android apps in practice, there exist three officially supported instruments, namely Accessibility Scanner (AS),^2^Lint,^3^ and Node Tree Debugging (NTD).^4^

The former takes a snapshot of an application as input. It scans each GUI component to identify accessibility issues related to content labels, touch target size, clickable items, and text/image contrast. The tool is based on dynamic analysis; therefore, it requires the app under investigation to be installed on a device. Lint is instead based on static analysis and runs as part of the Android SDK, even though it is also integrated within the Android Studio IDE. It has a broader scope than AS, as it reports micro-optimization opportunities to security, performance, and other non-functional aspects of source code. Lint also operates in terms of accessibility/usability and can detect problems related to missing content descriptions and accessibility labels. Finally, NTD is a testing tool for Android apps that can be employed to test for accessibility concerns. In particular, the tool describes how an AccessibilityService in the app interprets the GUI components and provides information as well as improvement recommendation related to focusable elements and their assistive descriptions.

It is also worth mentioning the existence of unofficial tools that are not integrated within the Android SDK but can provide developers with additional insights into mobile app accessibility. One of the most popular tools in this category is Enhanced UI Automator Viewer (Patil et al. 2016): it extends the standard UI Automator in order to verify unlabeled UI elements and color contrast.

Our work has an empirical connotation and, therefore, does not aim to improve the capabilities of the above-mentioned tools directly. Nevertheless, we provide pieces of information concerning accessibility guidelines and developers’ takes that are actionable for both tool vendors and researchers. The former can exploit them to tune the available tools, while the latter can devise novel approaches that better assist practitioners. We elaborate on these points when distilling the concrete implications of our work in Section 5.

## Research Methodology

The *goal* of our empirical study is to understand the state of the practice of accessibility in mobile applications, with the *purpose* of providing an overview of how mobile developers currently deal with this problem as well as the issues and challenges they face when implementing accessibility guidelines. The *perspective* is of both researchers and tool vendors. The former are interested in gathering insights into the current state of the practice on accessibility to devise novel possible instruments to support mobile developers when dealing with accessibility in practice. The latter are interested in tuning and providing new features that might further assist developers in assessing and improving accessibility aspects in mobile apps.

### Research Questions

We structure our investigation around two main research questions (**RQ** s). In the first place, we seek to understand how the existing accessibility guidelines are implemented in mobile applications, namely the extent to which developers adopt these guidelines when developing their apps. This leads to our first **RQ**: 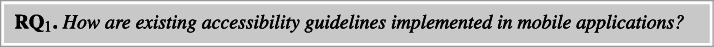


Once established how the accessibility guidelines are implemented, we proceed with a finer-grained understanding of developers’ perspective regarding the problem, particularly collecting their opinions on (i) the issues and challenges of implementing accessible applications and (ii) the tools currently supporting them. An improved understanding of those aspects would allow the research community to understand the developer’s needs to support further. Hence, we pose our second **RQ**: 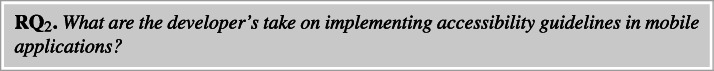


To address our **RQ** s, we conducted mixed-method research (Creswel 2009), combining manual coding analyses with surveys and semi-structured interviews with developers (Rossi et al. 2013). It is important to note that the empirical study has an *exploratory* connotation and, as such, it must be seen as an investigation whose outcome produces a number of implications that further research can exploit to generate research hypotheses.

### Material and Objects

The *objects* of the empirical study are represented by (i) mobile applications and (ii) accessibility guidelines.

As for the former, we focus on the 50 top-rated Android apps coming from the AndroidTimeMachine dataset (Geiger et al. 2018), which collects a reliable set of real open-source Android apps. We focus on these apps for two main reasons. On the one hand, we seek to analyze popular apps used by thousands, if not millions, users worldwide: this allows us to verify the behavior of developers who should be more sensitive to accessibility issues given the number of users they can potentially attract. On the other hand, we have to limit the number of applications to consider because of the time- and effort-intensive manual activities that we need to perform to address our research questions (further details in this respect are discussed later in Section 3.4).

As for the latter, Table 1 reports the entire set of accessibility guideline categories currently available for the design of Android applications. Each category groups a set of guidelines to account for when considering a specific aspect of the mobile application (e.g., *‘Audio and Video’*).

**Table 1:** Categories of the Accessibility guidelines for Android applications considered in the study

Guideline	Description
Audio and Video	When creating interactive content, consider font size, style/position
	of controls, and how content is presented. If there is a strong need for the
	content to auto-play, the user should be aware of it and be able to set
	preferences to prevent it.
Design	For the best user experience, aspects such as clarity on color contrast, color
	and meaning, touch target sizes, content resizing, actionable elements,
	visible focus, content consistency, and adjustability should be properly
	designed.
Editorial	Use of consistent labeling for buttons, links, and headings. Work closely
	with editorial colleagues to maintain consistency.
Focus	How content is visually presented can impact the order in which content is
	coded and, subsequently, the content order and focus order in which a
	user experiences the content, especially users with alternative input
	methods such as keyboard or screen reader users.
Forms	Provide labels for all form inputs and ensure form layout and order are clear.
	Related form inputs should follow each other, and, if needed, the visual
	design should be applied to imply grouping.
Images	Avoid the use of images of text and those that do not covey key information
	solely through a background image.
Links	Design content layouts that facilitate grouping text and images as one link.
Notifications	Design notifications to be inclusive and perceivable by all users. Where
	appropriate, include other feedback and assistance cues and prompts that
	might guide or encourage a user when needed.
Scripts and	Work from a basic core experience and progressively enhance this for more
dynamic content	capable users.
Structure	The design of the interface should convey the intended structure of the
	content. Identify headings, containers, and landmarks, working closely
	with UI/UX designers if needed.
Text Equivalents	The design of the non-textual content should describe their intent and not be
	used to convey meanings.

For the sake of understandability, we report in Table 2 a list of all the guidelines and their description. The identified accessibility guidelines are a set of technological agnostic best practices for mobile web content, hybrid, and native apps. The guidelines are based on the content requirements of three de-facto standard providers of information on the matter, i.e., the Android developer’s documentation,^5^ the World Wide Web Consortium (W3C) community,^6^ and the BBC Standards and Guidelines academy.^7^ We combine the three providers to create a comprehensive set of accessibility guidelines organized into 11 categories. Some guidelines are marked as *‘MUST’* or *‘MUST NOT’* (highlighted in and in the Table 2, respectively) depending on whether their implementation must be ensured or avoided. These guidelines are associated with specific, objective criteria that can assess their presence in a mobile app and can be implemented using the available mobile device technologies. As an example, audio-only or video-only content MUST be accompanied by a text transcript. Indeed, audio-only or video-only content would not be available to users who cannot hear or see, respectively. Transcripts of the audio and/or video must be provided as equivalent to allow users with disabilities to use and interact with an application properly. On the other hand, audio MUST NOT be played automatically unless the user is warned and can control the audio. This can be indeed harmful to users relying on assistive technologies such as speech output software to hear the page content, i.e., the auto-play feature would enable multiple audios at the same time, hence not allowing the user to properly interact with the app.

**Table 2:**
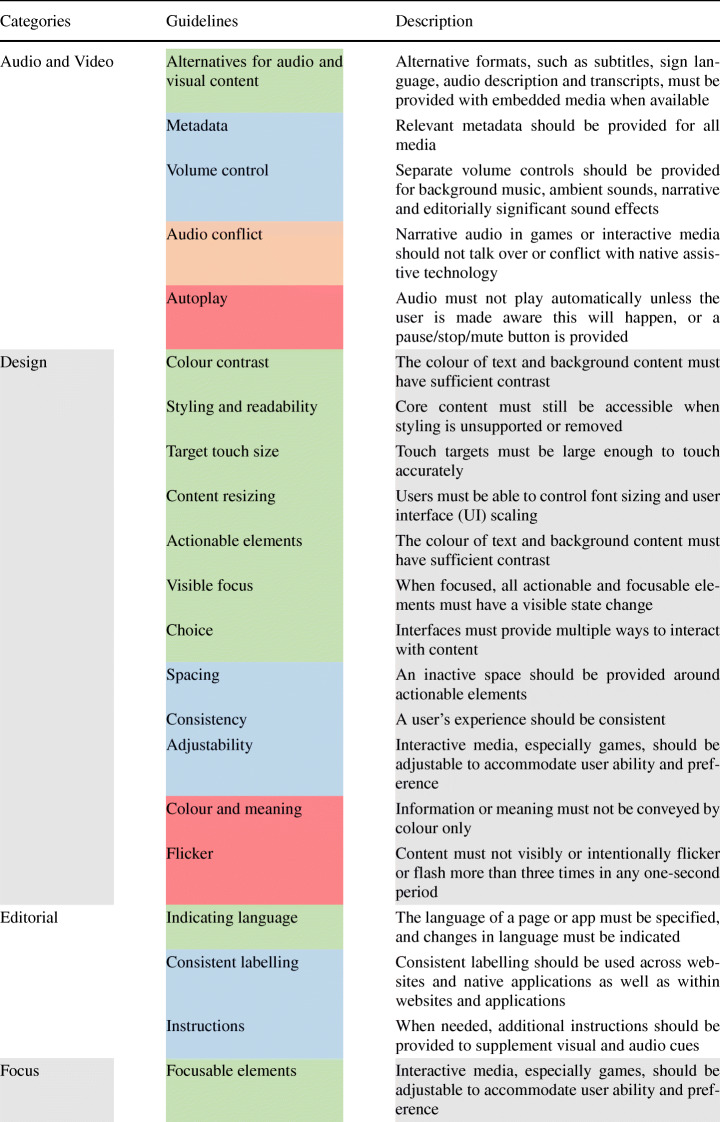

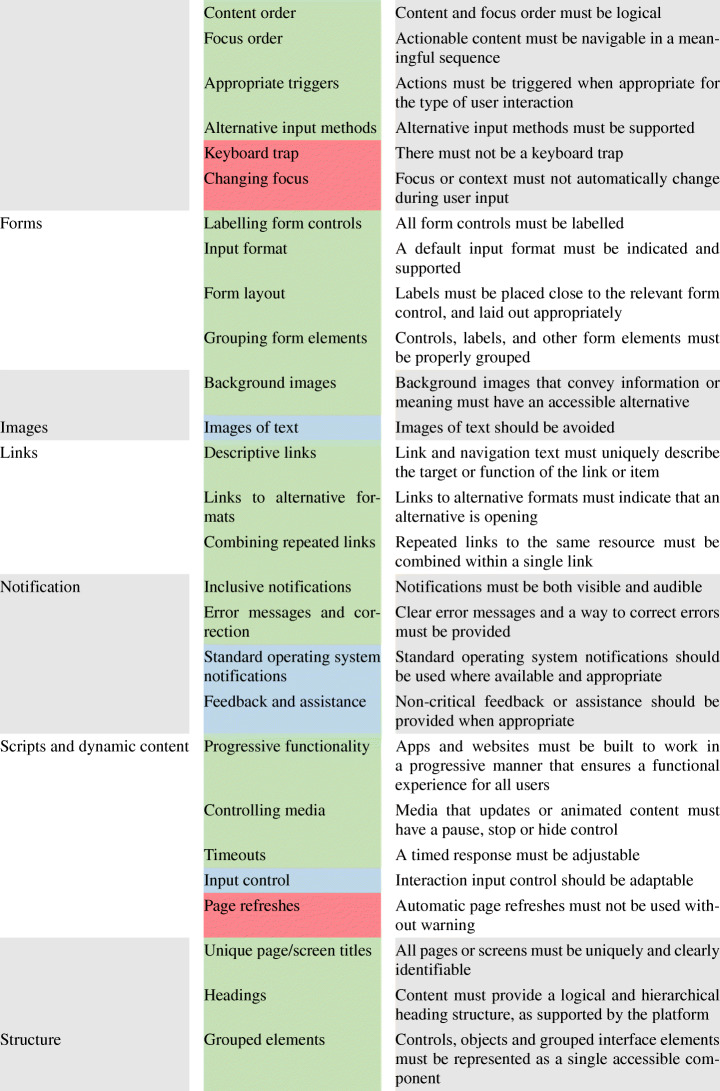

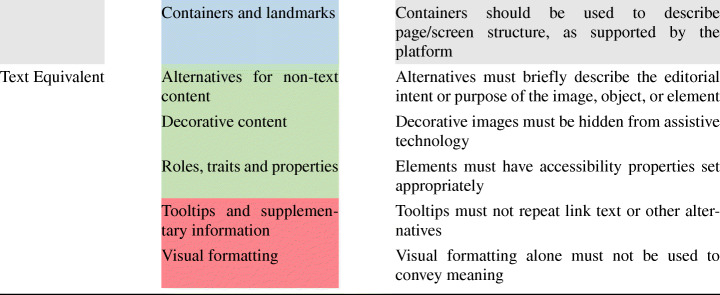
Accessibility guidelines for Android applications considered in the study

Other guidelines are marked as *‘SHOULD’* or *‘SHOULD NOT’* (highlighted in and in the Table 2, respectively): these represent less critical, yet important accessibility principles that should or should not be implemented. These guidelines are generally less testable and can be more subjectively interpreted by a user. For instance, separate volume controls should be provided for background music, ambient sounds, narrative, and editorially significant sound effects. Instead, narrative audio in games or interactive media should not talk over or conflict with native assistive technology.

### Subjects

The *subjects* of the study are developers of Android applications. We have involved both original and external developers of the applications that are the objects of the study. While the former can provide us with feedback on implementing the accessibility guidelines in their applications and their view of the problem, surveying a larger population of developers may provide additional insights into the issues and challenges of dealing with accessibility in practice. We collected participants’ background and demographic information to understand the representativeness of our results. We followed the sampling strategies defined in literature (Topp et al. 2004) to define a sample that meets our goals. More details on the recruitment strategies applied in our empirical study are reported in the next section.

### Execution of the Empirical Study

In this section, we report the methodological details that describe the execution of the empirical study—we discuss the two **RQ** s independently.

#### RQ_1_. Accessibility Guidelines in Practice

To address **RQ**_1_, we manually tested the considered applications to verify the implementation of accessibility guidelines—this strategy allows us to interact with an app and its accessibility services directly, much like a user would normally do. Overall, the guidelines to be verified were 54, divided into the 11 categories presented in Table 2. To perform such a manual test, we adopted a closed-coding strategy (Vaughn and Turner 2016): this is a systematic methodology that, in our case, involves the analysis of all the graphical user interfaces of an application and the subsequent labeling of the guidelines implemented as functionalities of the app, starting from a pre-established coding scheme, which is our case is represented by the set of guidelines available for Android applications.

More specifically, we have created a data extraction form, implemented using an Excel sheet, to facilitate the verification of the guidelines. For each of them, the form contains four pieces of information: (i) the name of the guideline to verify, (ii) the procedure to follow to discover whether the guideline is implemented, e.g., activate the notifications to verify that they are both visible and audible, (iii) the excepted visual/audio effect to observe in case the guideline is implemented, and (iv) the outcome to add once evaluating the guideline. The extraction process of an app was conducted by the first author of this paper and consisted of the following steps:

**Step 1—Download.**: The author downloaded the app from the Google Play Store on a Huawei Y5 smartphone.
**Step 2—Guideline identification.**: The author selected the next guideline to test and the corresponding instructions from the data extraction form.
**Step 3—Activation of accessibility features.**: Depending on the selected guideline, she has activated the accessibility function required to verify it if needed. Otherwise, she has gone straight to the next validation step.
**Step 4—Element identification.**: The author has been exercising the app to identify the feature connected to the accessibility guideline, if available. For instance, this concerns the identification of the app’s media in case the guideline refers to *‘Audio and Video’* accessibility aspects. If identified, the author proceeded with the next step; otherwise, she went back to Step 2 and continued with another guideline.
**Step 5—Verification of the guideline:** Once the element is identified, the author determined if the guideline is implemented in the app. If so, she annotated the data extraction form by putting, in the row corresponding to the considered guideline, a *‘true’* in the fourth column. Otherwise, she annotated the column with *‘false’*.

Using the above-described methodology, we have collected 50 Excel sheets, one for each application considered. These were later analyzed to address the first research question.

#### RQ_2_—Surveying Mobile Developers

To address **RQ**_2_, we conduct a survey study aiming at gathering insights regarding accessibility concerns from a broad audience of Android developers. The survey is composed of three main sections—we report the full list of questions in Table 3. The first one presents a total of nine questions about accessibility and how developers consider it in practice. We ask questions on the relevance of the problem, i.e., how important is accessibility for the participants, what reasons would make them willing to implement accessibility features in their applications, and whether they are aware of the existence of guidelines to make an app accessible. Afterward, we continue with questions more related to the implementation of accessibility guidelines. In particular, how often developers implement them in their applications, how difficult they are to apply, and why. Finally, we ask participants to report up to five challenges they usually face when dealing with accessibility concerns and report whether and which tools they currently use when performing the task.

**Table 3:** Full list of survey questions

n.	Question	Evaluation criterion
Section I. Accessibility of Android applications.		
1	In your opinion, how relevant is the problem	Likert scale from 1 (Not at all) to 5 (Very
	of accessibility?	important)
2	Please, tell us more about your answer.	Open answer.
3	What makes you willing (or not) to implement	Multiple Choice—it includes the *‘Other’*
	accessibility guidelines?	option.
4	To what extent are you aware of the accessibility	Likert scale from 1 (Not at all) to 5 (Very
	guidelines available for Android applications?	much)
5	To what extent do you follow accessibility guidelines	Likert scale from 1 (Not at all) to 5 (Very
	when developing Android applications?	much)
6	Can you please rate how difficult it is for you to	Likert scale from 1 (Not at all) to 5 (Very
	implement the following guidelines?	much) for each guideline.
7	For each guideline rated by the participant with	
	3/4/5 to question #6:	
	7.1. Can you please explain more about what makes	Open answer.
	it harder for you to implement the guideline?	
8	What are the top 3 problems of dealing with	Open answer.
	accessibility in Android development?	
9	What are the top 3 to 5 challenges you face when	Open answer
	dealing with accessibility concerns?	
10	Do you use any tool to verify the implementation	Open answer
	of accessibility guidelines?	
Section II. Further opinions.		
11	If you have further comments on the accessibility	Open answer.
	of Android applications and how you deal with	
	the problem, feel free to comment more on it.	
12	If you would like to receive a summary of our	Open answer.
	research results, please leave your e-mail.	
13	Would you be willing to participate in a follow-up	Yes/No.
	interview to better discuss the problem of	
	accessibility in Android development?	
Section III. Background.		
14	What is your current job?	Multiple Choice—it includes the *‘Other’*
		option.
15	What if your gender?	Multiple Choice—it includes the *‘Other’*
		option.
16	How do you rate your expertise with	Likert scale from 1 (Very poor) to 5 (Very
	programming?	high).
17	How do you rate your expertise with Android	Likert scale from 1 (Very poor) to 5 (Very
	programming?	high).
18	What is your company size?	Multiple Choice—it includes the *‘Other’*
		option.
19	What is your team size?	Multiple Choice—it includes the *‘Other’*
		option.

In the second part of the survey, we allow participants to provide us with additional insights and feedback. They can leave their e-mail address if they are interested in receiving a summary of our findings and can express their consent to a follow-up interview to discuss the problem of accessibility in practice further. sFinally, the third section of the survey concerns background information that we collect to understand better our sample of developers and possibly analyze the generalizability of our results.

The survey is designed to last 15/20 min and is created using a Google survey module. Before releasing the survey on a large scale, we ran a pilot with two developers of our contact network to evaluate if the survey is short and understandable enough to reduce the risk of having a low response rate and be appropriately filled out. Based on the pilot results, we have changed the text of some questions, add/remove some of them, or change the response type to make the questionnaire easier to understand or quicker to be compiled.

To gather insights from the original developers, we extract the e-mail addresses from the Github repositories of the considered applications. Then, we invite developers to fill the survey out, first asking whether they would like to participate. In other words, we recruit only volunteers to avoid privacy issues or other developer concerns. In addition, to gather insights from external mobile developers, we advertise the online survey using the personal social network accounts of the authors (i.e., Facebook, Twitter, and LinkedIn). It is worth remarking that we were aware that the reliance on social media might negatively impact selecting a valid sample. Therefore, we integrated social media with other sources to ensure the quality/completeness of the information gathered when addressing **RQ**_2_, still relying on a large sample of developers for our study. On the one hand, we involved additional developers from our private contacts (e.g., former University students or other practitioners who are currently mobile developers). On the other hand, we advertised the survey on a specialized practitioners’ blog such as Reddit lto acquire information from developers who have a solid knowledge of programming (Vassallo et al. 2020). In particular, Reddit contains more than 100 different subreddits dedicated to Android development that we exploit to potentially reach thousands of Android developers. We track the source used by participants to access the survey to better comment on the validity of the sample. To further stimulate the participation, we allow participants to indicate a non-profit organization of their choice to which we would donate 2 USD for the research against COVID19.

The answers are anonymized to preserve the privacy of participants. As a result of this study, we have a clearer view of the relevance of accessibility in practice and the major challenges developers face when dealing with the problem. Based on the answers received to question #13, we also planned follow-up semi-structured interviews with Android developers. Their main goal is to clarify ambiguous or contrasting answers received during the survey and to have a better picture of the current practices, issues, and challenges experienced by developers when dealing with accessibility in Android environments. From a practical perspective, we summarize the survey results to the interviewees and ask them to comment on the answers from which we could not derive a definitive outcome. The semi-structured interviews are conducted through Skype, last 30/40 min, and are transcribed for further analysis.

### Data Analysis

Once we gathered data from the closed-coding exercise and the survey study, we proceeded with their analysis.

As for **RQ**_1_, we first provided descriptive statistics on the extent to which accessibility guidelines are implemented in the sample of Android applications. We computed minimum, mean, median, standard deviation, and maximum number of accessibility guidelines implemented in the considered apps. Secondly, we provided a finer-grained overview of each specific category of guidelines. We discussed (i) to what extent each of them is present in the sample by reporting descriptive statistics, i.e., minimum, mean, median, standard deviation, and maximum number accessibility guidelines for each category, and (ii) the relative and absolute frequency of implementation of the guidelines included in each category. Then, we focused on the guideline requirements, i.e., *‘MUST’*, *‘MUST NOT’*, *‘SHOULD’*, and *‘SHOULD NOT’*: in this case, we aimed at understanding whether developers take them into account, e.g., if the *‘MUST’* guidelines are implemented in the considered apps. Finally, we verified the relation between the guidelines and the type of application considered. We grouped the apps by category, as provided by the Google Play Store, and we computed descriptive statistics to grasp if some categories are more prone to accessibility concerns.

As for **RQ**_2_, we first described the background of survey participants by discussing the answers they provide in Section III of the survey. This detail allowed us to understand the sample of developers and reason about the generalizability of our findings. In the second place, we distinguished the analysis procedures to use when considering closed and open questions. The former was analyzed employing statistics: we plotted and discussed the distribution of answers provided by participants through the Likert scale evaluations. The latter was subject of an *iterative content analysis*: in particular, we conducted the following methodological steps:

**Step 1—Microanalysis.**: The first author of the paper went through the content of the participant’s answers and the possible semi-structured interviews. She split sentences using standard text separators (e.g., commas) and assigned initial labels to each sentence: these labels represent the main concepts discussed by participants. Then, the three authors not involved so far validated the initial labels assigned and provided feedback on how to improve them, for instance, by proposing to aggregate two semantically similar labels. When this step was accomplished, we computed a measure of agreement between the labels assigned by the first author and those recommended by the other three.
**Step 2—Categorization.**: The first author used the suggestions and feedback received in the first step to conduct a second iteration over the labels assigned. This step resulted in a set of themes deemed important by participants when addressing each survey question.
**Step 3—Saturation.**: All the authors were involved in reaching a final agreement concerning the names and meanings of each label. This step led to a *theoretical saturation*, i.e., the point in which no further labels are required because the existing ones already correctly represent the concepts expressed by the study participants.

The themes coming from this data analysis procedure concern each specific open question posed in the survey. We discussed each theme and provided qualitative insights by presenting the most significant answers for a specific theme. In addition, when analyzed the answers to questions #8, #9, and #10 of the survey, we also provided statistical data reflecting the number of times a specific issue/challenge/tool named by the participants, hence providing a kind of prioritization of the concerns and tools that developers have concerning the problem of accessibility.

### Verifiability and Replicability

The data generated from our study are made persistently publicly available through Figshare (Di Gregorio et al. 2021). In particular, we release raw data about the accessibility guidelines implemented in our dataset, the survey structure, the anonymized responses, and all scripts used for data analysis.

## Analysis of the Results

This section presents the results of the empirical study, which we discuss by addressing the two research questions independently.

### RQ_1_. Accessibility Guidelines in Practice

In the context of **RQ**_1_, an iterative manual verification was performed to evaluate which accessibility guidelines were implemented within the mobile applications that are the subject of the study. As explained in Section 3.4, these were evaluated for their general applicability verifying whether each guideline was implemented or not in the application.

According to the results obtained, we observe that no application implemented all the guidelines. This result was somehow expected, other than reasonable, since the accessibility guidelines do not represent fixed rules. Their applications must therefore be considered based on the specific application domain and context. Nonetheless, we noticed that most of the guidelines were applied at least once in our dataset: as such, we can report that the mobile developers of the considered apps sometimes take care of them.

Looking deeper into the considered apps, we observed that 94% of the guidelines (51/54) could be assessed, i.e., the apps contained features that might have enhanced through the implementation of accessibility mechanisms. Other guidelines were instead not applicable. For instance, this is the case of the *‘Metadata’* guideline, which cannot be currently applicable in Android. Indeed, it does not support a mechanism for navigating between containers within native applications. A user can only navigate through a single item at a time. As a consequence, the *‘Containers and landmarks’* guideline is also not applicable. Finally, Android does not provide tooltips or additional hint text other than aria:contentDescription. Therefore, the *‘Tooltips and supplementary information’* guideline is not applicable—however, users can still obtain tooltips by long-pressing on icons in the Action Bar.

Based on the considerations above, we considered the number of guidelines that could be assessed while measuring the total amount of guidelines implemented within the considered applications. For instance, let consider the Budget application. In this case, 30 guidelines were assessable and, among these, ten were violated (i.e., 1/3 of them).

**Fig. 1:**
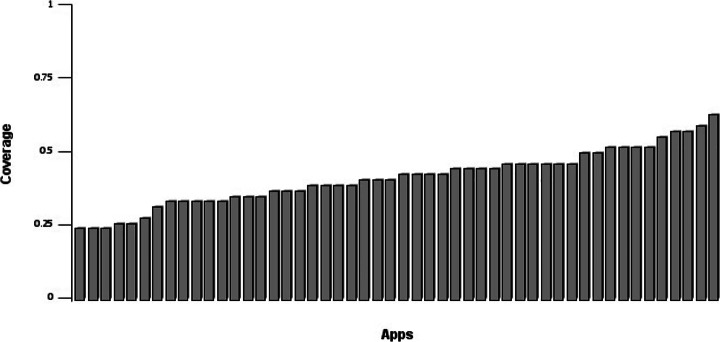
Guidelines coverage of the 50 apps. A coverage (y-axis) equal to 1 means that all guidelines were implemented in the analyzed apps

Figure 1 shows the percentage of guidelines implemented by the developers of the 50 apps considered. In particular, the x-axis represents apps (i.e., each bar is an app) while the y-axis reports their accessibility coverage level (i.e., the percentage of guidelines implemented). From the figure, we could immediately understand that the number of accessibility guidelines implemented in the considered apps was typically low, with a minimum of 24% and an average of 41%. In the best case, 63% of the guidelines were implemented. Consequently, we could first conclude that, overall, mobile developers tend not to implement accessibility guidelines while developing and maintaining their apps, even though they might have the chance to do that.

**Fig. 2:**
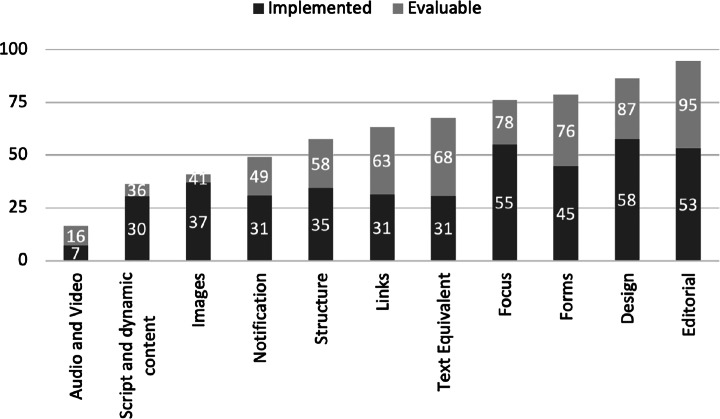
Percentage of guidelines assessable against percentage of guidelines actually implemented in the considered mobile applications, grouped by category

Figure 2 provides a more detailed overview of which are the individual accessibility guideline categories implemented in our dataset.

From the figure, we could observe that some guideline categories seem to be more considered by developers. For instance, the *‘Images’* category showed the highest ratio between the number of accessibility guidelines implemented and those actually assessable (37/41). This category refers to evocative visual content that allows the user to interpret the meaning of the features implemented in the applications. The highest implementation ratio is somehow reasonable and expected since the use of images to reflect the content of a piece of text is something that human beings typically do to convey meaningful messages (Turkle 2011). As such, independently from the knowledge that developers might have of the specific guidelines ruling the usage of images, we could have expected to observe the category to be highly implemented.

The category having the highest ratio of guidelines violated was *‘Audio and Video’* (only 44% of the guidelines were implemented over the total assessable). This indicates that mobile developers do not often take care of the characteristics that the interactive content should have in terms of font size, style/position of controls, and so on. In this case, it is likely that developers are not keen nor aware of the need to put themselves in the others shoes and offer functionalities that facilitate users to interact with the app.

As for the other guideline categories, we could delineate a general trend from the analysis of Fig. 2. A notable percentage of guidelines were violated: while we could not speculate on the reasons making them less implemented at this stage, we sought to understand this aspect further in the context of our second research question.

When lowering the granularity of our investigation to the individual guidelines within each category, we could first observe that the highest amount of guidelines implemented pertained to *‘Design’*, with an average of 28.8 out of a maximum of 50, i.e., around 29 applications contained implementation of accessibility guidelines related to the design of the application. More specifically, the *‘Style and readability’* design guideline, with a value of 47, appeared to be the most implemented, followed by the *‘Spacing’* guideline with a value of 45. On the contrary, the least evaluable category was *‘Audio and Video’*, which was also the least implemented with an average of 3.6. In this case, the *‘Volume control’* guideline was implemented only two times out of 50 applications.

**Fig. 3:**
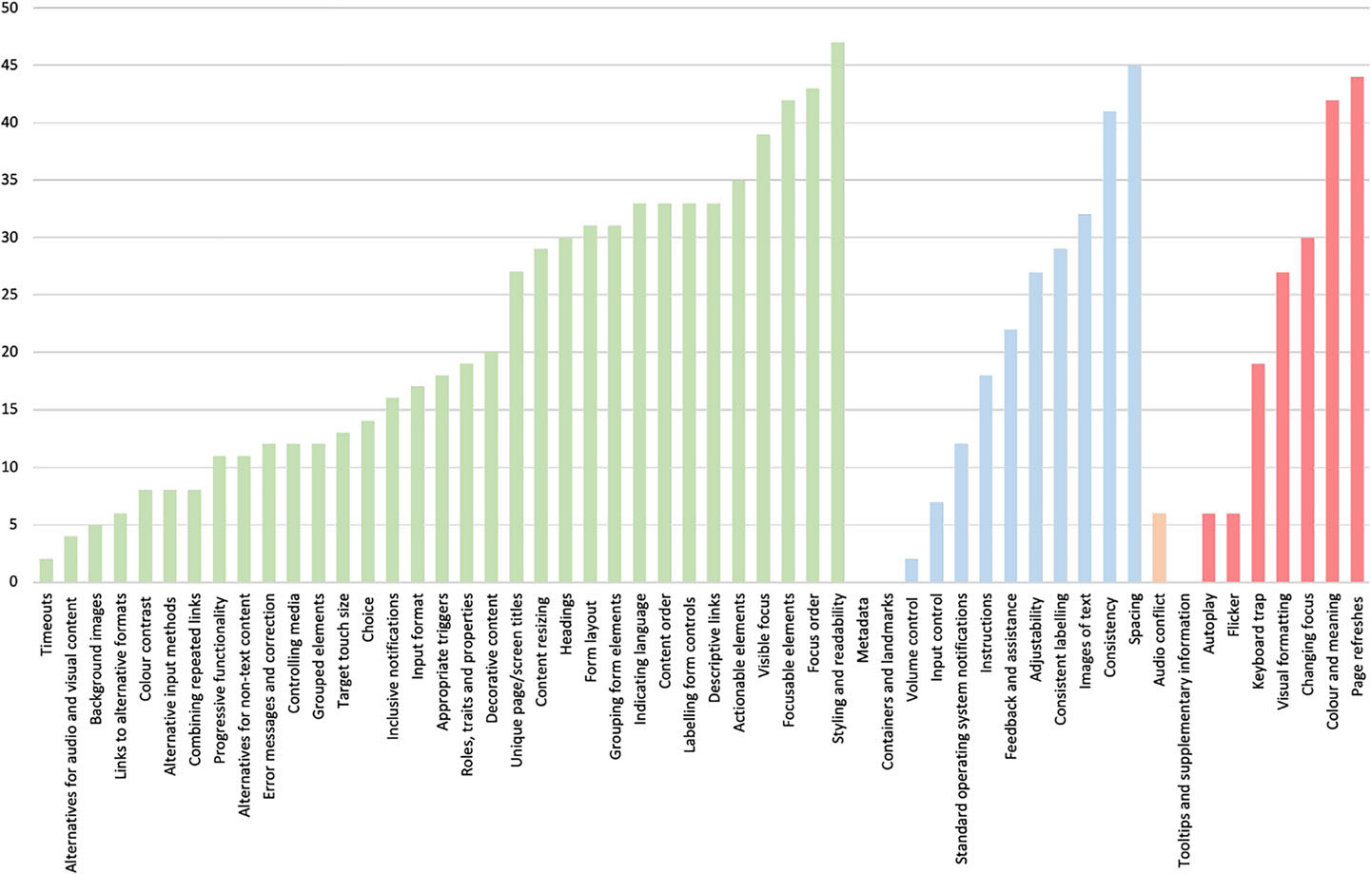
Distribution of accessibility guidelines implemented, grouped by requirements

When it turns to the guidelines requirements, i.e., *‘MUST’* , *‘MUST NOT’* , *‘SHOULD’* and *‘SHOULD NOT’*, Fig. 3 shows the distribution of guidelines applied grouped by their associated requirement—we visualize the distribution in ascending order based on the number of guidelines abide by. Such an assessment was intended to understand whether developers consider the guideline requirement while deciding which accessibility guidelines to apply. However, as depicted in the figure, we could not find any relation between those requirements and the application of the guidelines, meaning that developers do not likely consider whether a certain guideline must/should or not be applied. This aspect is further considered in our **RQ**_2_, where we inquired mobile developers on their expertise on accessibility concerns.

To conclude the discussion, from the first research question we could observe that developers of the top mobile applications considered often tend not to implement accessibility guidelines. This result is not connected to whether the guideline must/should (not) be applied. In our discussion, we also identified possible reasons behind the way developers operate in terms of accessibility. The next research question aims to elicit directly from the developer’s experience the main problems and challenges they face when dealing with the problem of accessibility of mobile applications., with the ultimate goal of deriving concrete actionable items and take-away messages that researchers and practitioners might consider to further investigate and address the problem of accessibility in mobile applications. 
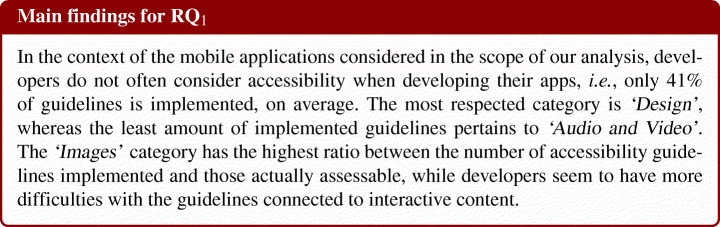


### RQ_2_. The Developer’s Take on Accessibility

While the previous research question allowed us to understand, from a quantitative perspective, how accessibility guidelines are implemented in Android applications, we could only delineate some conjectures on the reasons why developers decide to apply or not specific guidelines. The survey study conducted in **RQ**_2_ aimed at shedding light on the developer’s perspective of the accessibility problem. In particular, our survey was answered by a total of 75 developers, of which 63 male, four female, one transgender person, and seven who preferred not to declare it. Given the nature of the dissemination mechanisms, we cannot estimate the response rate—we are not aware of how many potential developers were reached over the various social networks and blogs considered. Nonetheless, we can report that 65% of the participants had access to the survey via personal contact, 11% via Telegram, 9% via Reddit, 8% via Facebook 5% via Twitter and 1% via Tandem.

#### Developers’ Background

**Fig. 4:**
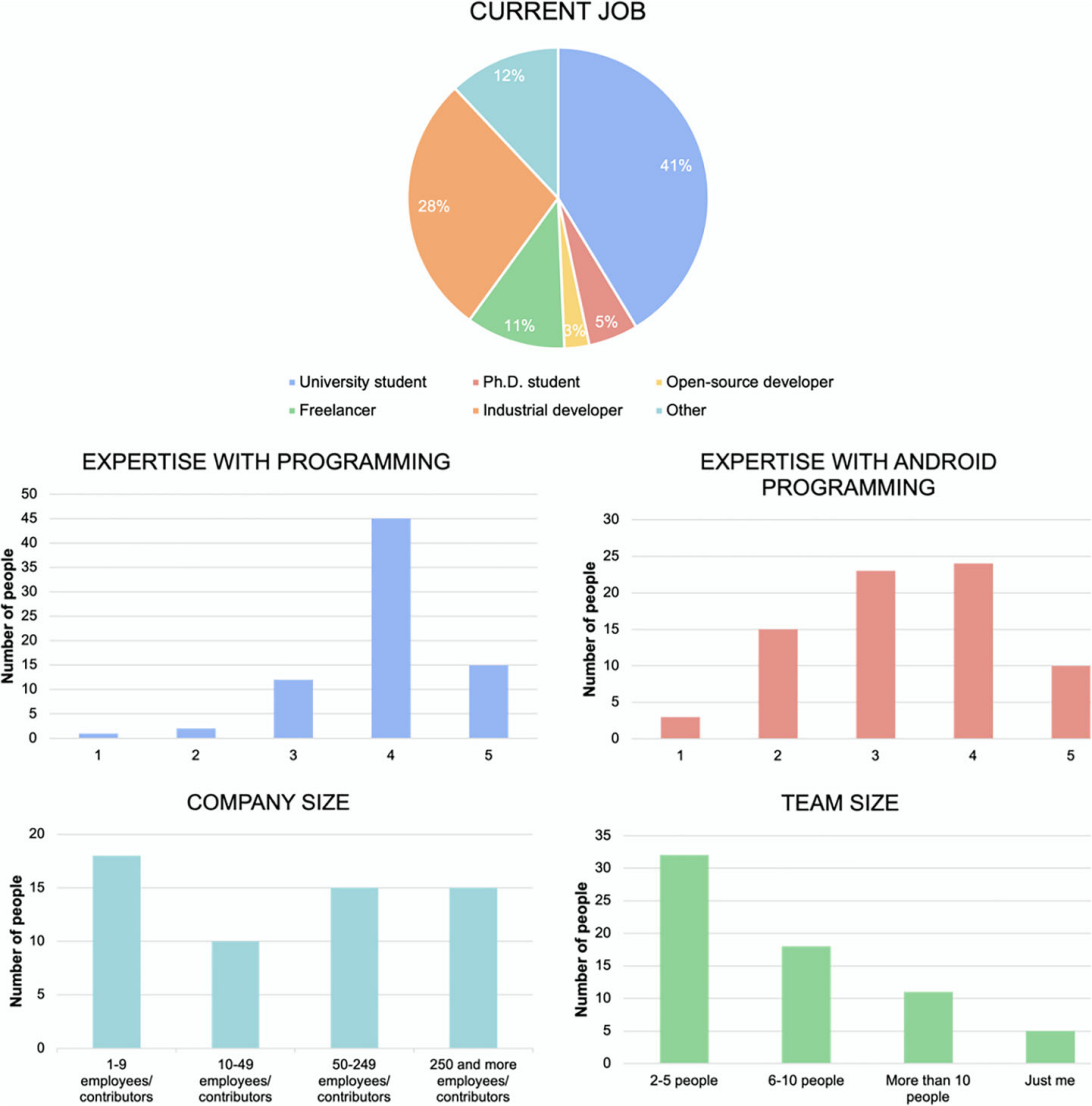
Background of our participants

Figure 4 shows the background of our participants. Among the 75 respondents, 60% (45 participants) declared to have a high level of experience in programming, and 43% (32 participants) had high experience in Android programming. About 42% of the participants (mainly) work as developers and 24% (18) work in medium-sized companies with more than 100 employees. From these descriptive statistics, we can say that our sample consisted of various developers with sufficient programming experience and whose opinions may provide us with valid and reliable insights on how they deal with the accessibility problem. Furthermore, 15% of participants work in a large team of 10 to 200 people, 24% within a team of 5–10 people, while the majority (43%) in a small team (i.e., 2–5 people).

#### Relevance of the Problem

In the first part of the questionnaire, we aimed at understanding how developers consider accessibility in practice when developing mobile apps.

**Fig. 5:**
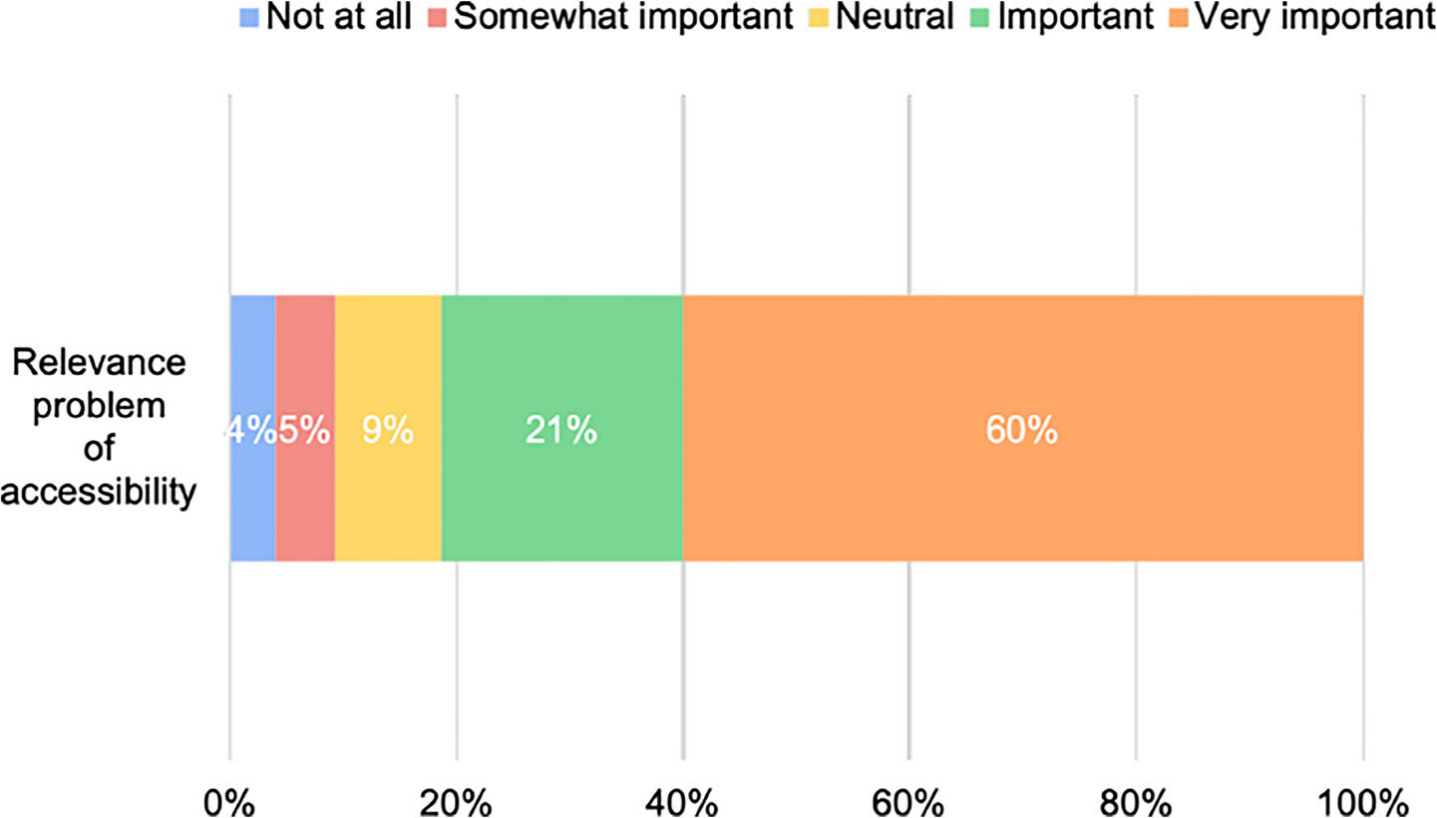
Relevance of the accessibility problem from the developer’s perspective

Figure 5 shows how many participants responded with values between the minimum and maximum to the first question of the survey. As shown, most of the participants (60%) considered the problem of accessibility as *very important* for mobile app development. At the same time, only three developers (4% of our sample) claimed that this is negligible. Hence, as expected, we can confirm that accessibility is a significant concern for most of the developers involved in the survey. As an example, one of the participants commented:

#26 -*All users need to have the same possibilities.*

The high perceived relevance of the problem allows us to claim that accessibility is definitively something that researchers should further explore with the aim of providing automated support or even empirical studies that may increase the developer’s awareness about the problem.

#### Reasons for Implementing (or Not) Accessibility Guidelines

**Fig. 6:**
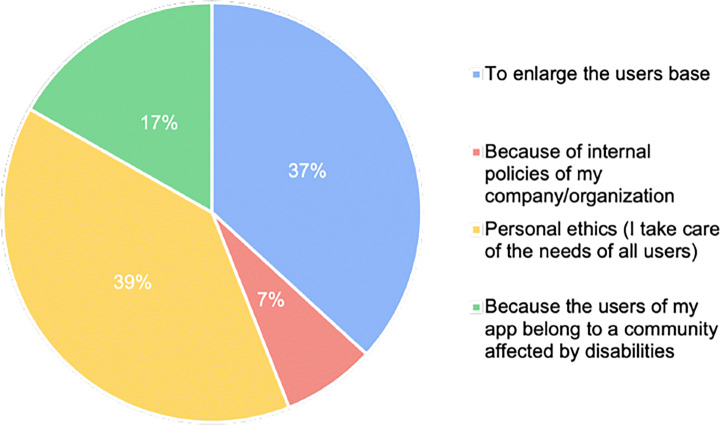
Results for Question n.3—what makes you willing (or unwilling) to implement the accessibility guidelines

**Fig. 7:**
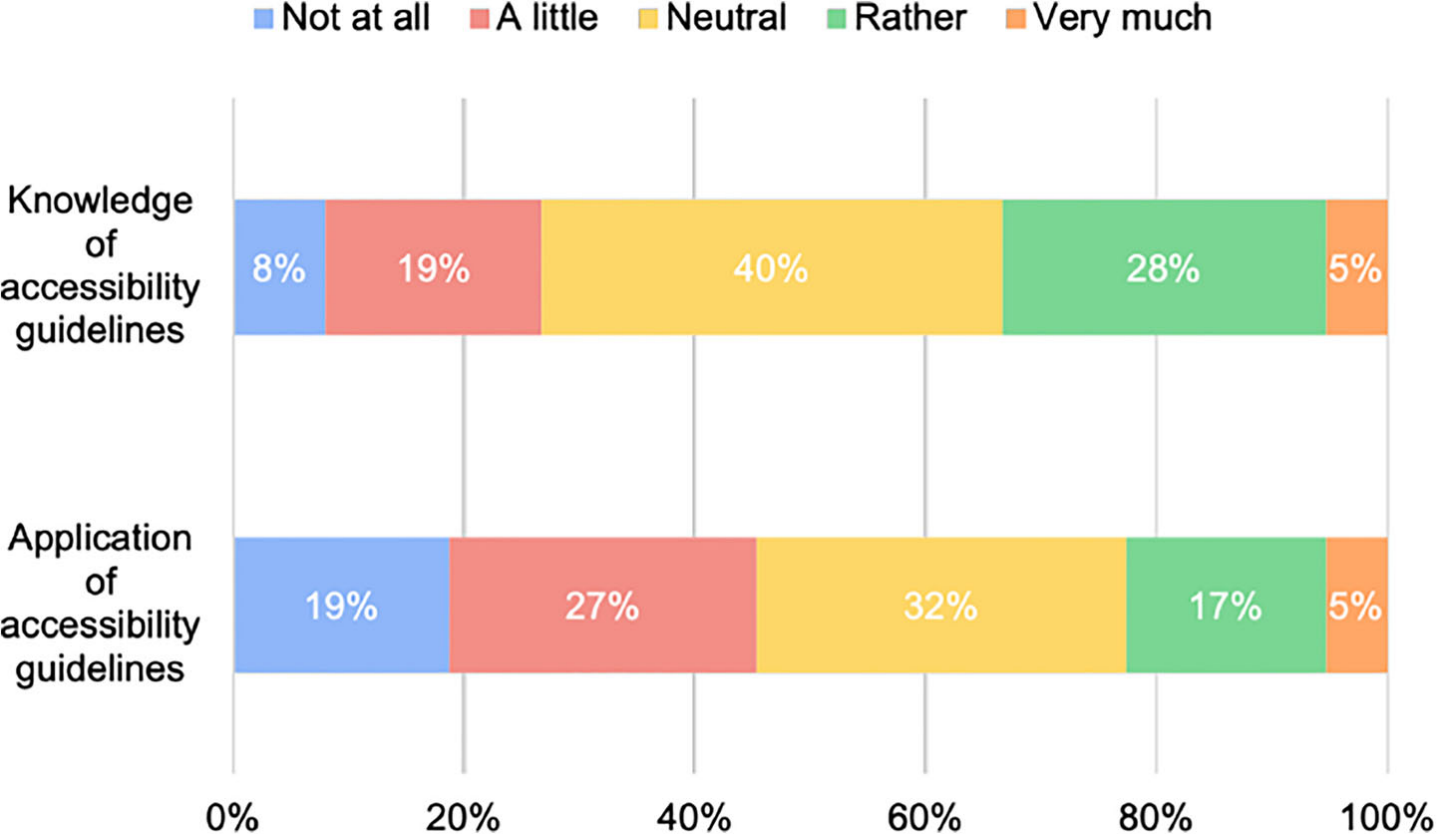
*Results for Questions n.4 and n.5*—awareness and implementation of accessibility guidelines

Personal ethics (39%) and the widening of the potential user base (37%) were mentioned as the main reasons making developers willing (or unwilling) to implement the accessibility guidelines, as depicted in Fig. 6. A smaller percentage of participants (17%) declared that their applications are dedicated to people with disabilities and, therefore, they have to follow accessibility guidelines. Only 7% of the developers reported that their companies implement policies aimed at ensuring mobile accessibility. Looking at these results, we can observe that the main driver for the implementation of accessibility guidelines is the personal willingness of developers to provide additional functionalities that would enable the usage of the app to a wider variety of users. By matching these observations with the poor implementation of accessibility guidelines discovered in **RQ**_1_, our findings seem to suggest that more work should be done on motivating developers and stimulating their willingness to apply accessibility guidelines while developing their apps. This result is confirmed by the analysis of questions #4 and #5 of our survey (Fig. 7): although most of the participants have a medium-high knowledge of accessibility guidelines, a large majority of participants apply them only in a few cases.

While the results of our analysis mainly report on the need for making developers aware of the relevance of accessibility, they might be also read under an orthogonal point of view. According to the opinions collected, the developers who typically apply accessibility guidelines do that because of personal motivations, which are by nature connected to their degree of sensibility. Hence, besides raising awareness of the problem, developers might benefit from additional instruments such as, for instance, improved advertisement strategies on the relevance of accessibility in practice and how a lack of it might impact the life of people with disabilities. In this respect, we could envision a multidisciplinary effort conducted by multiple stakeholders.

#### The Opinion on the Individual Categories

**Fig. 8:**
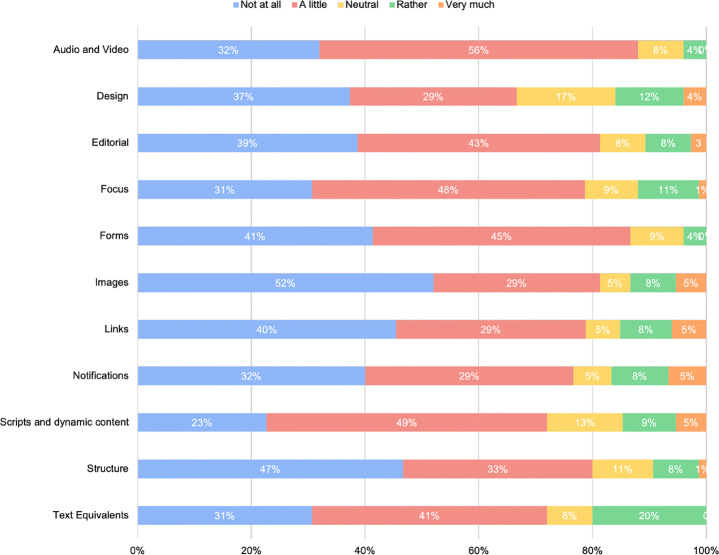
Results Questions n.7 and n.7.1—difficulty in implementing the specific guidelines

Once investigated the general behavior of developers, our survey aimed at seeking their opinions and experience with the implementation of the individual categories of guidelines. Figure 8 reports the results obtained in this respect. It is worth remarking that our survey allowed participants to express additional opinions on the factors making hard the implementation of specific guidelines. While the comments left were helpful in most cases, others were not clear enough to elicit those factors. In these cases, we took advantage of the follow-up semi-structured interviews conducted with the participants to discuss them further. In particular, eight developers left their email addresses in response to question #13 of our survey and were later interviewed. For the sake of readability and conciseness, we discuss the results by guideline, reporting data from the survey and accompanying the discussion with the insights coming from the semi-structured interviews whenever needed.

In the first place, our analysis revealed that most developers encounter little or any difficulty in implementing the guidelines. As a matter of fact, for the *‘Audio and Video’*, *‘Forms’*, and *‘Text equivalent’* guidelines, no developer found it very difficult to implement the category of guidelines. This result was quite surprising and, at the same time, interesting: while the involved developers consider the vast majority of accessibility guidelines as easy (or fairly easy) to implement, they are still reluctant to implement them—hence, confirming that the problem is strictly connected to the willingness or, perhaps, a limited understanding of how significant might be implementing those guidelines for users with disabilities. However, there are some exceptions.

##### Design

Diving deeper into the individual guideline categories identified by at least one developer as hard to implement, three participants declared the *‘Design’* category to be a difficult one. From the survey analysis, we found that developers mostly focus (or need to focus) on the aesthetics of the application rather than on its accessibility. As such, they would need more precise guidelines for implementing the design principles that address accessibility concerns. This consideration is also common to other developers, who commented, for instance, saying that:

#23 -*Standards are not defined precisely.*

The semi-structured interviews confirmed that the guideline definitions are sometimes vague and not easily interpretable, potentially complicating their implementation. For this reason, Interviewee #3 claimed that an improved accessibility guideline should provide informal definitions and concrete examples on how to integrate them within various types of applications. This tooling would help developers to learn by examples, simplifying and speeding up the implementation process.

##### Editorial

82% of the participants considered the implementation of the guideline to be not very difficult or not difficult at all. The remaining 14 developers, instead, rated this guideline as complex or very complex to apply. By looking at the open comments left by those participants, we could understand that the guideline is not complex. However, the time required for implementing it is too high and/or there is a lack of resources available. Two developers commented, indeed, that:

#57 -*A lot of businesses just don’t have enough resources to comply with all such consistency across all clients.*

#63 -*It is very tedious and takes a significant amount of time to label interactive elements and images.*

##### Focus

The vast majority of the participants did not consider this guideline hard to implement. Only one participant justified the complexity of the implementation by saying that:

#17 -*I saw many examples of buggy focusability in android development and sometimes providing for example good keyboard navigation on the screen is really really hard due to these bugs.*

In other words, the developer suggested that the Android APIs to use for implementing this guideline may sometimes be defective, increasing the time and effort required to ensure the focusability of the app. Once again, this seems not to be strictly connected to the guideline itself but to the surrounding environment required to implement accessibility guidelines.

##### Images

13% of the participants reported that ensuring the accessibility of images can be hard or very hard. By looking at the open answers provided, we could understand that this is mainly due to the role played by images in the graphical user interface of mobile applications. One of the developers commented as follows:

#56 -*Difficult as the images play an important graphic role.*

Unfortunately, the comment could not provide us with a clear understanding of the key issues connected to the implementation of the guideline. For this reason, we have further elaborated the question in our follow-up interviews with the developers. From the discussion, it turned out that this is due to the lack of proper usability skills, which might lead to complex implementations of this guideline. Indeed, optimizing the use of images while keeping accessibility under control is not easy, as implementing the guidelines risk affecting the overall aesthetics and look-and-feel of the app, potentially creating more issues than benefits. This result suggests that the definition of recommendation approaches that may suggest how to best implement the GUI of mobile apps by balancing usability and accessibility might be a nice addition for mobile developers.

##### Links

This category presents a very similar situation as for the previous guideline, with a lower percentage (40%) of users who did not rate the implementation difficult. Nonetheless, we were surprised to see some open comments like the one shown below:

#72 -*Didn’t even know.*

By discussing this further in the semi-structured interviews, we understood that some developers were not even aware of the existence of this guideline. Interviewee #6 explained that most of the developers with whom s/he worked were not only unaware of accessibility guidelines but also unable to find helpful information on the web. As a result, the implementation difficulty is sometimes due to the retrieval of appropriate information on usability and accessibility, making it hard for developers to correct the problem in their apps.

##### Notifications

As shown in Fig. 8, 5% of the respondents to our survey (4) declared that implementing notification-related accessibility guidelines is hard in practice. In this case, the comments left in the open answers were already clear enough to elicit the main reasons behind this result. One of the participants stated:

#70 -*Notifications in Android are often very finicky and device-dependent so we can’t expect them to conform reliably to specific behaviors.*

As reported, the mechanisms enabling notifications in Android are not always easy to use. This challenge may be related to the different notification types that can be visualized differently, and developers should apply the guidelines to the specific implementations. Among all the guidelines discussed so far, this seems to be the most source code-related one: in this sense, the definition of smart mechanisms may potentially address the problems raised by the participants, e.g., dynamic wizards helping developers to select the most appropriate set of notifications along with the accessibility rules to implement.

##### Scripts and Dynamic Content

As for this category, 14% of developers rated its implementation as fairly or very difficult. As stated by one of the developers, the implementation of this guideline:

#64 -*Would harm the simple user interface, adding effort and making the development of simple apps unfeasible.*

As reported, some developers would not find enough benefits from the implementation of this guideline, as it may have possible negative effects on the aesthetics and usability of the app. While discussing this issue further in the semi-structured interviews, Interviewee #4 commented on the statement above by discussing the exemplary case of *Progressive Functionality*. This fine-grained guideline is related to creating graphical user interfaces that allow users to do actions in a stepwise, progressive manner. S/he reported that:

Interviewee #4—*The fundamental problem with progressive content is that it takes what was previously one requirement (do x when user enters the screen) and turns it into three or four requirements (do x or do y or do z based on condition A B or C). These types of multi-requirements are actually quite difficult to communicate about (conversations will be full of confusion and miscommunication). Naturally they increase the workload but if the requirements are clear it is actually not all that much work. The problem is getting the requirements clear in the first place. Additionally, it can be very difficult for the quality team to actually exercise all of these different pathways, so it increases the work there too. Most developers are going to advise against these type of progressive interfaces and instead promote that you create one interface that works for all situations, perhaps with the ability to have some content hidden by default.*

In other words, the implementation of scripts and dynamic content enforces developers to increase the number of app requirements, requiring them to create more test cases or develop more code review activities. In addition, the definition of the requirements might be a source of miscommunication that possibly leads to the introduction of undesired defects. Based on our results and discussion, implementing this guideline seems to be related to multiple aspects and levels of expertise covering the entire software life cycle. As such, developers might be more reluctant to consider its actual application.

##### Structure

The last guideline we discuss is concerned with the structured content. More particularly, one participant reported that:

#74 -*Sometimes it is difficult to maintain the original structure and refactoring is required.*

This answer was later discussed in the semi-structured interviews. The main point here is concerned with the moment in which accessibility is considered. If an application is not designed to be accessible in the first place, refactoring for accessibility can be effort-prone and costly since it may imply the re-design of entire screens of the app. In addition, Interviewee #7 pointed out the lack of automated mechanisms and integrated tools that can provide accessibility feedback directly within the IDE. In her opinion, the availability of these tools might help to address the problem of accessibility from the start, hence avoiding costly refactoring that is rarely implemented.

#### The Challenges of Accessibility

The last part of our survey was reserved for the issues and challenges of accessibility in practice. Table 4 reports the top-5 list of challenges identified when analyzing the participants’ answers.

**Table 4:** 5 top challenges to face when accessibility problems are encountered

n	Challenge
1	Raise awareness of accessibility and the needs of disabled users.
2	Standardize accessibility guidelines during app implementation.
3	Implement accessibility guidelines without compromising the
	aesthetics and functionality of the application.
4	Involve more disabled users during application development.
5	Raise awareness of companies and customers on the accessibility
	problem of universal inclusion.

As shown in the table, two of these challenges are related to accessibility awareness. In the first place, developers expressed their inability to understand the exact needs of users with disabilities: this represented the main, most popular challenge mentioned in our survey. Participants also reported that one of the challenges concerns the involvement of those users during the development: this is made complicated by identifying the right target audience and the mechanisms to use for involving them. For example, developers mentioned the complexity of requirement elicitation, which naturally leads to ineffective solutions. Our participants (and our interviewees) suggested using a user-centered methodology to develop mobile apps, where real users are surveyed and involved throughout the application development process.

At the same time, our participants mentioned the awareness of companies and customers. When discussing this further, the developers told us that only a small percentage of users need accessibility in mobile applications and, therefore, companies tend to underestimate the problem. In addition, accessibility is often considered non-portable and essential only for large apps. Perhaps more importantly, all interviewees raised another social issue connected to accessibility: they argued that their customers sometimes dictate not to follow accessibility guidelines to get the product up and running in the least amount of time. Consequently, they found it difficult to convince the customers of the additional time required to implement a universally usable product.

Additional challenges are more on the technical side. On the one hand, standardizing accessibility guidelines is related to defining techniques that help developers implement the guidelines while the app is still under development: accessibility should be considered a first-class citizen. On the other hand, developers need mechanisms that allow for a trade-off between the aesthetics of the graphical user interface and its accessibility.

As further elaborated in Section 5, the challenges identified impact the mobile application development from requirement elicitation to low-level design and implementation, other than letting emerge important socio-technical implications of accessibility. In the first place, there exist communication barriers that prevent developers to engage with disabled users. The redesign of current requirement elicitation strategies seems therefore to be the next reasonable step to pursue. The definition of new communication channels that might allow users with disabilities to advertise their needs, the creation of accessibility interest groups, or even the definition of regulations and policies that rule the certification of mobile apps with respect to accessibility requirements would be the next big challenges for practitioners, researchers, and decision makers. At the same time, our results seem to suggest the need for novel continuous validation and verification mechanisms that would reduce the development effort when dealing with accessibility. In this sense, the definition of user-centered methodologies that may put users with disabilities in the loop would provide additional opportunities for developers to get in contact with minorities and account for their opinions and constant support when evolving mobile apps.

#### The Current Accessibility Support

As a final step of the survey, we asked participants if they use tools to verify the implementation of the accessibility guidelines. 77% of the respondents do not use tools. The remaining 13% reported the usage of the Accessibility Scanner app, the Google Play pre-launch report, and the definition of beta tests with users. Our results clearly show that it is not very common for developers to rely on tools to verify the accessibility of the apps being developed. Three of the respondents reported that the missing usage of tools is because they provide minimal information while lacking a more detailed and careful analysis of both the different categories of disabilities that should be considered and the specific guidelines that should be implemented. In addition, the interviewees highlighted that different types of devices must be taken into consideration; often, different brands/models of devices behave differently. Therefore, the implementation of some accessibility UIs requires complex logic to include all target devices. Last but not least, some brands’ battery-saving policies may affect or even suspend accessibility services.

The results coming from this analysis point out the need for further automated support from the software engineering community. We believe that our findings might be especially interesting for the software testing perspective: The developer’s answers indeed revolve around the verification of how accessibility guidelines are implemented, other than the compatibility concerns that arise when the accessibility guidelines are implemented on multiple devices. 
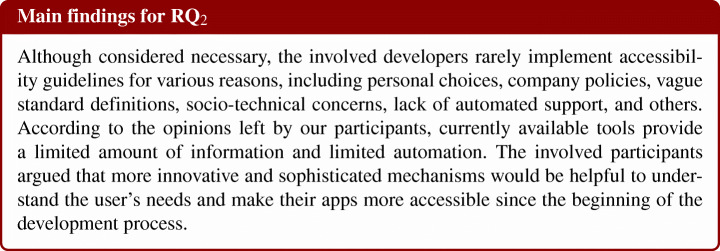


## Discussion and Implications

The results achieved when addressing our research questions provided several insights that need to be further discussed and implications for both researchers and tool vendors, which we elaborate on in the following.

### Conclusion 1—Accessibility Problems are Widespread in Apps

One of the main results coming from our analyses refers to the poor adoption of accessibility guidelines in practice. We discovered that, in each app, most of the guidelines were not considered by developers in the implementation phase or incorrectly applied. Furthermore, our results show that *‘Design’*, *‘Script and Dynamic Content’*, and *‘Text Equivalents’* are the most problematic categories of guidelines to implement. The feedback received by developers through surveys and follow-up semi-structured interviews allowed us to elicit the main reasons behind such difficulties. We conclude that several technical aspects connected to the implementation of accessibility guidelines should deserve further attention in the future. On the one hand, more research is needed around this subject. We hope that the reported results might serve as a basis for stimulating software engineering researchers to proactively consider novel mechanisms to support mobile developers. A critical challenge here concerns the definition of (semi-)automated techniques that might support developers while evaluating the level of accessibility of their applications and developing accessible mobile apps. On the other hand, the lack of standardized methods to apply accessibility guidelines represents a challenge for the designers of these guidelines. While there exist catalogs that suggest the general universal design principles to apply, the results of our study pointed out the lack of practical recommendations and patterns to follow during the development. This latter challenge is, in particular, one of the main points of our future research agenda: the definition of *accessibility design patterns* will be the next challenge to face.

### Conclusion 2—Lack of Developer’S Awareness of Different Types of Disabilities

Not only the problem of accessibility is widespread in practice, but also developers generally lack knowledge of the different disabilities of users and how they should be considered from a software engineering perspective. This clear issue represents a call to action for researchers working in multiple fields, from medical branches to software engineering and human-computer interaction. Indeed, the results of our study revealed the need for multidisciplinary research that can formulate novel instruments and methods to increase the sensibility of developers around the matter. This finding also highlighted the lack of software engineering education and training on accessibility and the need to review the existing guidelines of software engineering college curricula to focus more broadly on accessibility as a quality attribute and be considered throughout the software development lifecycle. Accessibility is typically mainly taught in Human-Computer Interaction courses within computer science education. Our findings may instead stimulate discussions on how to improve study plans in order to incorporate accessibility differently, for instance by adding new courses or remodularizing existing ones. Along this line, Waller et al. (2009) have recently proposed an educational approach where accessibility is not treated as a separate topic but rather as an integral part of software design and development. We can envision even further adjustments like the definition of accessibility requirements in greenfield software engineering projects developed by students at both Bachelor and Master levels, so that students can engage with accessibility within the more complex design of software projects and possibly take decisions driven by the accessibility requirements fixed. In a similar way, we can envision the integration of accessibility modules within courses of Mobile and Web Development or Basic and Advanced Programming. As pointed out by Jia et al. (2021), these requirements could instill in students greater awareness of accessibility in programming topics without affecting the learning objectives of basic computer science. Last but not least, educators should consider the various forms of disability when training the next generation of mobile developers, trying to convey and teach the principles of Universal Design. For example, students could analyze and discuss case studies to learn methods to design, implement, and test accessible systems to assemble fully inclusive systems, even when the target audience is not restricted to disabled users.

### Conclusion 3—Software Engineering Meets Human-Computer Interaction

As pointed out by several developers involved in **RQ**_2_, the accessibility perspective of the mobile engineering process is all but defined. Developers argued the difficulties they experience with accessibility requirement elicitation and management, other than the challenges concerning design, refactoring, and testing for accessibility. Besides the actions that developers might take individually, e.g., the user-centered usability testing processes mentioned above, our findings are more general and recall the need of *software engineering methods for accessibility*. This aspect is one of the main implications of our work. We argue the definition of symbiotic methods that would allow human-computer interaction and pure software engineering to more closely collaborate to define unified processes that may enable improved engineering of mobile applications that take accessibility and usability into account. The rise of a tighter collaboration between the two research communities would enable the definition of combined methods that optimize the quality of mobile apps simultaneously with usability, possibly leading to the production of better software.

More specifically, we believe that our findings have implications for both software engineering methodology and practice. In the former case, we argue the definition of a brand new branch of software engineering specifically focused on human-computer interaction methods for accessibility (and usability in general). This encompasses the entire software engineering life-cycle. We first envision novel methods to manage the management and development complexity of accessibility guidelines. On the one hand, this recalls the need for understanding the orthogonal expertise that mobile app developers require to properly deal with the matter or even how developers can interact with designers or UX experts. Researchers working at the intersection between project management and social debt might be interested in assessing how such mixture of expertise can be managed or lead to sub-optimal communication/collaboration practices, other than exploring the many ways these practices may impact the development and commercial success of mobile apps. The results reported in our paper are also of the interest of researchers and agencies working on the definition of standards: the guidelines available are indeed not yet transferred into suitable standards nor manuals that can be practically used by developers. In this respect, our findings open the way to other developer-centered investigations into how accessibility standards can be devised and integrated within the developer’s workflow.

From a technical side, software maintenance, evolution, and testing researchers are critically affected by our findings. Among the various points raised by developers in the context of **RQ**_2_, we first identified traces of a new type of technical debt (Kruchten et al. 2012) connected to the management of accessibility concerns. In particular, one approach to mitigate accessibility issues is to plan for accessibility early in the design phase rather than managing it as an afterthought at the end of the development phase. In other words, our results allow us to define an *accessibility technical debt* as the accumulated long-term cost caused by choosing an early, sub-optimal user interface or design solution. So far, this unknown debt has been neglected by the research community. We argue that additional analyses and researches would be needed and desirable to devise new accessibility technical debt detectors and refactoring recommenders. These tools could work at different granularities (e.g., in the IDE rather than during code review) and stages (e.g., designing early prototypes, such as UI sketches, refactoring existing functionalities). In a similar fashion, we could envision novel catalogs of *accessibility design patterns*, that may support the practical implementation of novel standards. Last but not least, the lack of tooling reported by the involved developers multiple times in our analysis clearly open the door for verification and validation researchers interested in defining instruments to check and verify the source code for the presence of accessibility concerns.

### Conclusion 4—On the Generalizability of our Findings

The generalizability of the findings is a crucial aspect for any empirical investigation. In the context of our investigation, there are some observations to make in this respect. In the first place, when considering the accessibility guidelines implemented in practice, we could focus only the limited set composed of 50 top-rated mobile applications belonging to the AndroidTimeMachine dataset (Geiger et al. 2018) because of the intensive manual work required to address **RQ**_1_. We found that, on average, only 41% of the guidelines are actually applied in those apps. While the reader must deem these results valid within the specific context analyzed in our work and look for larger-scale replications of the study, the rationale behind the poor application of accessibility guidelines let us believe that the main findings of the analysis might be observed in other apps as well. The lack of awareness and tooling to deal with accessibility, along with the other reasons identified, may indeed limit the overall applicability of the guidelines, independently from the size or popularity of the apps. In this sense, we expect to discover similar findings when considering a larger sample of apps. Additionally, it is also worth remarking that our study targeted top-rated apps, which are supposed to take care of accessibility concerns with the aim of enlarging their user base. It is likely to believe that lower-rated apps follow a similar behavior to gather more and more users. In terms of generalizability of the findings reported in **RQ**_2_, the motivations provided by the sample of 75 developers look reasonable enough to believe they can be considered valid by other developers as well. At the same time, inquiring a different sample of developers might have let to the analysis of additional perspectives, thus potentially leading to the identification of more challenges/barriers to accessibility, other than less obvious/popular motivations not to implement guidelines. In this respect, the reader might only consider the set of observations and conclusions provided as partial. Replications of the survey study would indeed provide additional insights into the problem and, subsequently, the conclusions drawn on the status of accessibility.

## Threats to Validity

Several threats might have affected the validity of our results and the conclusions drawn. This section discusses how we mitigated them.

### Threats to Construct Validity

Possible issues in this category refer to the methods used to set up the empirical study. The first point of discussion concerns the dataset of mobile apps exploited in the study. We focused on 50 top-rated Android apps coming from the AndroidTimeMachine dataset (Geiger et al. 2018): the decision was made to collect and analyze data of real open-source Android apps that are used by thousands of users worldwide.

Another possible concern is connected to how we elicit the set of issues and challenges developers face when dealing with accessibility. We opted for a survey-based investigation through which participants could share their past/current experiences with the matter. Of course, those participants performed the task in a remote setting. While we could not completely avoid the lack of conscientious responses, the follow-up data analysis allowed us to verify the meaningfulness of the answers. It could have possibly detected data to be removed for the sake of reliability. In addition, we performed semi-structured interviews to complement the survey study and discuss questions for which we obtained contrasting outcomes.

### Threats to Conclusion Validity

In the context of **RQ**_1_, we conducted an iterative manual analysis to verify the presence of accessibility guidelines. Similarly, in **RQ**_2_, we conducted an additional coding procedure to analyze the developer’s open answers. In both processes, the first author of the paper was the main responsible. Nevertheless, to double-check her actions and mitigate possible misinterpretation, the other authors have constantly been involved and took action whenever needed. This continuous collaboration and the level of agreement reached make us confident of the results reported in the study. Although we cannot claim the statistical relevance of our sample across the entire Android ecosystem, the analysis depicts the status of accessibility in the best-rated open-source Android apps. We are, however, aware of the need of further replications or even complementary studies that might corroborate the conclusions drawn by employing our methodology.

### Threats to External Validity

Threats in this category refer to the generalizability of the conclusions drawn from our study. As already discussed in Section 5, our findings should mainly be deemed valid with respect to the sample of apps and developers considered. More specifically, we targeted 50 top-rated open-source mobile applications and involved 75 survey respondents. As for the former, the considered apps belonged to different domains and had various characteristics that enabled us to investigate accessibility under different perspectives. As for the latter, the participants had previous experience and expertise with Android development, hence being able to provide us with meaningful insights into the problem of accessibility. While some of the results identified in the context of our analysis look reasonable enough to be potentially considered applicable to other apps and developers, we cannot claim the generalizability of our results to mobile applications having a different connotation, e.g., closed-source or industrially developed apps. As such, further larger-scale replications of our study in different contexts would be desirable, other than already part of our future research agenda.

## Conclusion

The growing popularity of mobile devices, coupled with constant technological improvements in the field, has led to an increasing number of mobile applications. In this context, usability aspects play a pivotal role both when considering the design and implementation phases. Although usability is already recognized as a crucial aspect of mobile development, only a few studies analyzed the accessibility of mobile applications. In this research, we aimed at advancing the state of the art by analyzing (1) the extent to which a set of known accessibility guidelines are applied in practice and (2) the developer’s take on the accessibility problem.

We conducted a quantitative investigation of 50 Android applications finding that most of the guidelines available are not implemented within applications. Afterward, we interviewed 75 developers, conducting eight semi-structured interviews, showing that accessibility is perceived necessary, but several socio-technical barriers often prevent developers from applying the accessibility guidelines. The overall output of our research identified several challenges that must not only be considered by the software engineering research community but also by experts of other disciplines like human-computer interaction, medicine, and others.

The identified challenges represent the main input for our future research on the subject. We aim to further explore accessibility on a more extensive set of systems, possibly considering how the same application on different operating systems can generate a different level of accessibility. Perhaps more importantly, we will seek to elicit a set of accessibility design patterns that would enable developers to more practically deal with the accessibility guidelines and define novel automated instruments to facilitate the adoption of accessibility guidelines. Finally, we plan to enlarge the scope of our analyses to understand the app customer’s perspective, namely how the accessibility of mobile applications should be improved from the point of view of the users with disabilities.

## Footnotes

1 The registered report was accepted at the 2020 IEEE International Conference on Software Maintenance and Evolution (ICSME) and uploaded to the Open Science Framework (OSF); the report is available at https://osf.io/3yghp/.

2 https://support.google.com/accessibility/android/answer/6376570

3 https://developer.android.com/studio/write/lint

4 https://developer.android.com/guide/topics/ui/accessibility

5 https://developer.android.com/guide/topics/ui/accessibility/index.html

6 https://www.w3.org/TR/mobile-accessibility-mapping/

7 https://www.bbc.co.uk/guidelines/futuremedia/accessibility/mobile
